# 

**Published:** 1910-12-10

**Authors:** 


CONTENTS.

| | PAGE |
|---|---|
| **Women's Work in War** | 305 |
| **The Enteric Fever "Carrier"** | 306 |
| **Annotations**— A New Medical School in London—The Alleged Case of Covering—A New Form of Malingering | 307 |
| **Medical Opinion and Movement** | 308 |
| **Hospital Clinics**— The Prognosis of Cancer. By Dr. Reclus | 311 |
| **Special Article**— The Typhoid Carrier | 314 |
| **Diseases of Children**— The Early Recognition of Measles | 317 |
| **The Royal Army Medical Corps Section**— The St. John Ambulance Association and the Voluntary Aid Detachments | 318 |
| **Dermatology**— On the Use of Plasters in Dermatology | 319 |
| **Medico-Legal Points**— Workmen's Compensation Cases | 320 |
| **News and Events of the Week** | 321 |
| **Obituary** | 322 |
| **The Week's Work**— The Ideal Ward Floor—The Maple Floor—Further Points from the Report—Hospitals and their Medical Schools—Australian Hospitals and Old Age Pensioners The Royal Hospital for Incurables—This Week's Drug Market | 323 |
| **Special Institutional Articles**— Eminent Chairman Series. III.—Mr. Cosmo Bonsor, D.L. | 325 |
| **Parliament and Publications** | 328 |
| **Institutional Notes and News** | 329 |
| **Appointments and Resignations** | 330 |
| **Editor's Letter-Box**— Essex County Hospital | 330 |
| **The Hospital World** | 331 |
| **New Appliances and Things Medical** | 332 |
| **Jottings** | 332 |
| **Gifts and Bequests** | 334 |

